# Deletion of the CD2v Gene from the Genome of ASFV-Kenya-IX-1033 Partially Reduces Virulence and Induces Protection in Pigs

**DOI:** 10.3390/v14091917

**Published:** 2022-08-30

**Authors:** Johanneke D. Hemmink, Emmanuel M. Khazalwa, Hussein M. Abkallo, Bernard Oduor, Jeremiah Khayumbi, Nicholas Svitek, Sonal P. Henson, Sandra Blome, Günther Keil, Richard P. Bishop, Lucilla Steinaa

**Affiliations:** 1 Animal and Human Health Program, International Livestock Research Institute, Nairobi 00100, Kenya; 2 Deep Seq, School of Life Sciences, University of Nottingham, Nottingham NG7 2UH, UK; 3 Institute of Diagnostic Virology, Friedrich-Loeffler-Institut, Suedufer 10, Insel Riems, 17493 Greifswald, Germany; 4 International Livestock Research Institute, Nairobi 00100, Kenya

**Keywords:** African swine fever, CD2v, vaccine, gene deletion

## Abstract

Infection of pigs with the African swine fever virus (ASFV) leads to a devastating hemorrhagic disease with a high mortality of up to 100%. In this study, a CD2v gene deletion was introduced to a genotype IX virus from East Africa, ASFV-Kenya-IX-1033 (ASFV-Kenya-IX-1033-∆CD2v), to investigate whether this deletion led to reduced virulence in domestic pigs and to see if inoculation with this LA-ASFV could induce protective immunity against parental virus challenge. All pigs inoculated with ASFV-Kenya-IX-1033-ΔCD2v survived inoculation but presented with fever, reduced appetite and lethargy. ASFV genomic copies were detected in only one animal at one time point. Seven out of eight animals survived subsequent challenge with the pathogenic parental strain (87.5%) but had mild to moderate clinical symptoms and had a gross pathology compatible with chronic ASFV infection. All mock-immunised animals developed acute ASF upon challenge with ASFV-Kenya-IX-1033 and were euthanised upon meeting the humane endpoint criteria. ASFV genome copy numbers after challenge were similar in the two groups. ASFV-Kenya-IX-1033-∆CD2v is therefore a useful tool to investigate the development of immunity to ASFV genotype IX, but safety concerns preclude its use as a candidate vaccine without further attenuation.

## 1. Introduction

African swine fever virus (ASFV) is a large double-stranded DNA virus of the Asfarviridae family. It is responsible for the disease known as African swine fever, usually leading to an acute severe haemorrhagic disease with high mortality rates. However, some field strains induce mild or subclinical disease with low mortality. ASF is endemic in sub-Saharan Africa and Sardinia, with 24 genotypes detected in sub-Saharan Africa [1]. More recently, outbreaks of ASF caused by genotype II ASFV (referred to as the Georgia strain) have occurred in Eastern Europe, South-East Asia, China and the Island of Hispaniola [2]. Vietnam has issued a commercial permit for the use of a live-attenuated ASF vaccine (LA-ASFV) based on the Georgia strain [3], but the use of the vaccine is not permitted in the rest of the world.

Several other LA-ASFV strains with different gene deletions have been tested for virulence and protection against challenge with the parental virus strains, with several candidate LA-ASFV providing 100% protection against challenge with the parental strain [4]. The virulence of these LA-ASFV is reduced when used to infect domestic pigs due to the deletion of genes associated with virulence. The gene deletions can be induced either naturally (present in field strains), after prolonged cell culture, or introduced using genome editing techniques [5,6,7,8,9,10].

One of the candidate genes for gene deletion to reduce the virulence of ASFV is CD2v. The CD2-like protein, also known as CD2v, EP402R, or 8-DR, is responsible for the characteristic rosette formation of erythrocytes around ASFV-infected cells and binding of extracellular virus particles to erythrocytes, and plays a role in the immunomodulation of host responses [11,12,13,14]. It is the only protein confirmed to be present on the external envelope of the virus particle [15]. However, confocal microscopy has revealed that most of the expressed protein is expressed inside the infected cell rather than on the cell surface [14,16]. Some low-virulence ASFV isolates are non-haemadsorbing and have a truncated or deleted CD2v protein [17,18]. Furthermore, the experimental deletion of CD2v from the virulent BA71 genotype I strain led to a complete attenuation upon in vivo infection [19] and partial attenuation was also seen when CD2v was deleted from Benin∆DP148R [11]. In contrast, the deletion of CD2v (8-DR) in the Malawian strain Lil-20/1 did not change disease onset, disease course, or mortality, but a delay was seen in the onset of viraemia and there was a reduction in viraemia titres [12]. Similarly, the deletion of CD2v in the Georgia strain did not lead to a reduction in virulence in vivo and led to a limited reduction in viraemia [20]. Thus, the deletion of CD2v can have a different effect on the virus depending on the ASFV strain used.

As it is unclear whether an LA-ASFV based on a particular strain can protect against challenge with more distant ASF viruses, there is a need to develop LA-ASFV with the appropriate background for use in different geographical areas. This is particularly relevant for sub-Saharan Africa, where different genotypes are circulating simultaneously. A well-characterised genotype IX strain, ASFV-Kenya-IX-1033, is available that can be used for the generation of LA-ASFV with a genotype IX backbone [8,21,22]. In this study, a CD2v gene deletion was introduced to ASFV-Kenya-IX-1033 (ASFV-Kenya-IX-1033-∆CD2v) to investigate whether this deletion led to reduced virulence in domestic pigs, and to see if inoculation with this LA-ASFV could induce protective immunity against parental virus challenge.

## 2. Materials and Methods

### 2.1. Animal Experiments

The animal experiment was conducted in the ABSL-2+ facility at the International Livestock Research Institute (Nairobi, Kenya). Ethical approval was provided by the ILRI Institutional Animal Care and Usage Committee (IACUC2020-11). On arrival at the ILRI facilities, pigs were dewormed and vaccinated against foot and mouth disease and held in a quarantine facility for at least 21 days prior to the start of the experiment. Only healthy animals, which were seronegative for ASFV and which had no detectable ASFV genome copies prior to the start of the experiment, were included in the study. The pigs were crosses between Duroc and Large White, weighing between 45 and 70 kg at the start of the study.

**Figure 1:**
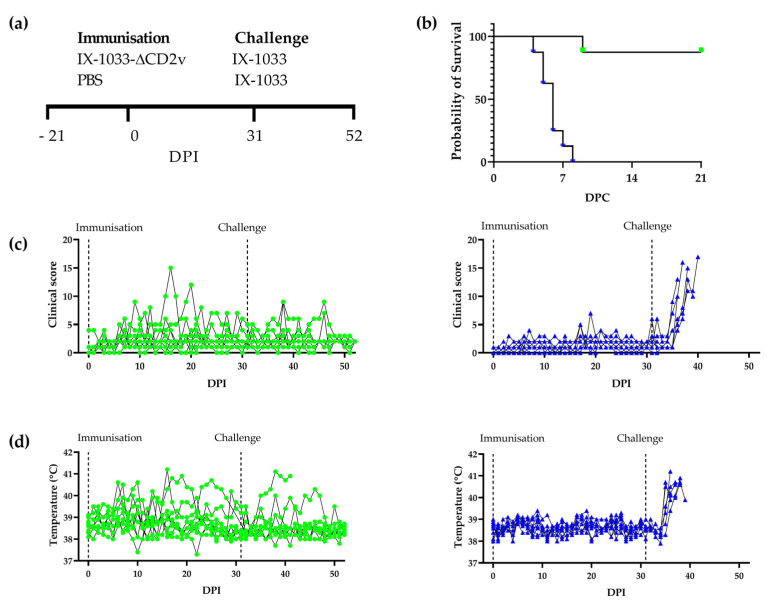
Experimental design, survival and clinical evaluation after immunisation and challenge. (**a**) Experimental design: pigs underwent quarantine for at least 21 days prior to the start of the study. DPI 0: pigs were immunised with ASFV-Kenya-IX-1033-ΔCD2 or PBS; DPI 31: pigs were challenged with ASFV-Kenya-IX-1033. The experiment was terminated on DPI 51. (**b**) Kaplan–Meier survival curve after challenge with wildtype ASFV-Kenya-IX-1033 in animals after immunisation with ASFV-Kenya-IX-1033-ΔCD2 (green) or PBS (blue) and challenge with ASFV-Kenya-IX-1033. Days post challenge (DPC). (**c**) Cumulative clinical scores in animals after immunisation with ASFV-Kenya-IX-1033-ΔCD2 (green) or PBS (blue) and challenge with ASFV-Kenya-IX-1033. (**d**) Rectal temperatures in animals after immunisation with ASFV-Kenya-IX-1033-ΔCD2 (green) or PBS (blue) and challenge with ASFV-Kenya-IX-1033.

Details on the experimental design can be found in Figure 1a. After the quarantine period, animals were assigned to their experimental groups and transferred to the ABSL-2+ facilities for acclimatisation. On Day 0, animals were inoculated by intramuscular injection in the neck with either 10^3^ TCID_50_ of ASFV-Kenya-IX-1033-ΔCD2v or PBS. One animal per group was euthanised on Day 27 and the remaining 8 animals per group were challenged on Day 31 with 10^2^ HAD_50_ ASFV-Kenya-IX-1033. Clinical signs, including rectal temperature, lethargy, anorexia, respiratory distress, vomiting, signs of skin haemorrhage and diarrhoea, were recorded as previously described [23]. Animals were euthanised when predetermined humane endpoint criteria were met or at the end of the study and PM investigation was performed. Titres of the stocks used for immunisation and challenge were confirmed by back titration. EDTA blood and serum were collected regularly for the determination of viral titres. Pieces of tissue were collected at PM for viral titre determination.

### 2.2. ASF Viruses for Immunisation and Challenge

Animals were immunised with 10^3^ TCID_50_ ASFV-Kenya-IX-1033-ΔCD2v. The construction of this virus is detailed in Hübner et al. [24] and in vitro growth and genome analysis of this virus is described in Abkallo et al. [8]. The titre of the stock was determined by TCID_50_ by observing fluorescent foci (dsRed) using fluorescent microscopy.

For challenge, 10^2^ HAD_50_ ASFV-Kenya-IX-1033 was used. Details on the virus, including whole genome analysis, are detailed in Hemmink et al. [21,22]. The titre of the stock was determined by HAD_50_ assay on pulmonary alveolar macrophages.

### 2.3. PBMC Isolation

Blood was collected from each animal in heparinised vacutainer tubes (Ref: 368480, BD). PBMCs were isolated using the Ficoll Paque^TM^ (Ref: 17.1440.03, GE Healthcare, Uppsala, Sweden) density gradient centrifugation technique. Briefly, the heparinised blood was mixed with an equal volume of sterile PBS, layered on Ficoll Paque and centrifuged at 1650× *g* for 30 min at RT with no brakes. The interphase layer containing PBMCs was collected, topped up with sterile 1xPBS and spun at 670× *g* for 10 min with brakes on. The pellet was resuspended in 10 mL of tris-ammonium chloride and incubated in a water bath set at 37 °C for 10 min to lyse residual RBC. The cell suspension was spun at 300× *g* for 10 min to remove residual platelets. The cell pellet was washed twice by suspending in 1xPBS and spun at 300× *g* for 10 min. After washing, PBMCs were re-suspended in complete RPMI1640 ((Ref: R4130-10L, Sigma) supplemented with 10% foetal bovine serum (Ref: A4766, GIBCO), 1% L-glutamine (Ref: 21051040, Thermo Fisher Scientific) and 1% penicillin/streptomycin (Ref: HP10.1, Roth).

### 2.4. IFN-γ ELISPOT

ELISPOT plates (Ref: MAHAS4510Millipore,) were coated with 100 µL per well of 1:1000 diluted mouse anti-pig IFN-γ antibody (clone P2G10 (Ref: 559961, BD Pharmingen)) in carbonate buffer and left overnight at 4 °C. The plates were washed five times using sterile PBS (200 µL per well) and blocked with 4% skimmed milk in PBS (Marvel original, Premier Foods group, Thame, United Kingdom) for 2 h at room temperature. The plates were washed five times with PBS, before adding 50 µL of antigens: Medium, Con A (10 ug/mL) or ASFV-Kenya1033-IX (MOI = 0.1). PBMC were seeded at 5.0 × 10^5^ per well, and plates were incubated overnight at 37 °C with 5% CO_2_.

Plates were washed five times with 200 µL per well of 0.05 % Tween 20 in PBS (PBS-Tween). Then, 100 µL of 1:2000 diluted biotinylated mouse anti-pig IFN-γ antibody (clone P2C11 (Ref: 559958, BD Pharmingen)) was added to each well and incubated for 2 h at room temperature. Plates were washed five times with PBS-Tween, before 100 µL of 1:1000 diluted streptavidin alkaline phosphatase (Ref: SA1008, Invitrogen) was added to each well and incubated at room temperature for 1 h. The plates were washed six times with PBS-Tween, and 50 µL of the SIGMAFAST TM BCIP^®^/NBT substrate (Ref: B5655, Sigma) was added per well and incubated for 20 min in the dark. The plates were washed with tap water to stop the reaction and then immersed in 1% formaldehyde solution for 10 min to inactivate residual viruses. Finally, the plates were washed in tap water and kept at room temperature to air dry. The spots were counted using the AID classic ELISPOT reader (AID AutoImmun Diagnostika GMBH).

### 2.5. Determination of Antibody Response

Blood was collected from each animal in serum-separating vacutainer tubes (Ref: 367837, BD) and centrifuged at 1500× *g* for 10 min and serum-harvested. ASFV anti-p72 antibodies were detected using the Ingezim PPA Compac Kit (Ref: 11.PPA.K3, Ingenesa, Madrid, Spain) according to the manufacturer’s protocol. Briefly, 50 µL of serum was added to the wells of the ELISA plate with 50 µL diluent to give a 1:1 dilution. Plates were incubated at 37 °C for 1 h, followed by 4 washes with wash solution, before adding 100 µL of diluted conjugate dilution to each well. Plates were incubated at 37 °C for 45 min and washed 5× in wash solution. Then, 100 µL of substrate was added per well and incubated in the dark for 15 min at room temperature before adding 100 µL of stop solution. Optical densities were read immediately at 450 nm wavelength using the Synergy HTX multi-mode reader (Ref: S12FA, BioTeK). Percentage of blocking was calculated according to the manufacturer’s protocol.

### 2.6. Determination of Viral Titres by p72/B646L qPCR

p72/B646L qPCR was used to assess the virus DNA content in EDTA blood and tissue samples. Genomic DNA was extracted from 200 μL of EDTA anti-coagulated blood using the Zymo Quick-DNA miniprep DNA extraction kit (Ref: D3025, Zymo research, USA). For the detection of ASFV genome copies in tissues, approximately 0.01 g to 0.025 g of splenic, submandibular and gastrohepatic lymph tissue was weighed and DNA was extracted using the Qiagen Dneasy Blood and Tissue Kits (Ref: 69506, Qiagen, Hilden, Germany). qPCR was performed as per the OIE-recommended real-time PCR assay according to King et al. [25], but primer and probe sequences were adapted to the Genotype IX challenge strain. Primer sequences were (P72-F ‘CTGCTCACGGTATCAATCTTATCGA’, P72-R ‘GATACCACAAGATCAGCCGT’ and P72 probe ‘FAM-CCACGGGAGGAATACCAACCCAGCG-TAMRA3’. The plasmid standard dilutions and qPCR conditions are described in Abkallo et al. [8].

## 3. Results

### 3.1. Clinical Signs after Immunisation

Animals were immunised by inoculation with ASFV-Kenya-IX-1033-∆CD2v or by mock immunisation using PBS and challenged 31 days later with the parental pathogenic ASF-Kenya-IX-1033 (Figure 1a). All pigs inoculated with ASFV-Kenya-IX-1033-∆CD2v survived for 31 days after immunisation, indicating that the virulence of ASFV-Kenya-IX-1033-∆CD2v was reduced compared to ASFV-Kenya-IX-1033, which induces 100% mortality within 4–7 days of inoculation [21]. However, the presence of mild to moderate clinical signs in all pigs demonstrated that the virus was not fully attenuated, and residual virulence was present (Figure 1c,d). Eight out of nine animals had fever (rectal temperature ≥39.5 °C) in the follow-up period after immunisation; this fever occurred at different time points and for different durations. Some animals had more than one period of fever. Six animals had a high rectal temperature (≥40.5 °C) for at least one day. In contrast, none of the pigs inoculated with PBS had a body temperature over 39.5 °C (Figure 1d). Lethargy was seen in seven animals immunised with ASFV-Kenya-IX-1033-∆CD2v, at different time points after immunisation and for different durations. Some animals had more than one incident of lethargy. In the PBS-inoculated group, two animals, which were treated for lameness, were less active and thus were scored as lethargic during the study. The remaining animals in the PBS group were all active after inoculation.

Joint swelling and lameness occurred in both groups, with joint swelling in all animals inoculated with ASFV-Kenya-IX-1033-∆CD2v, among which four pigs required treatment due to lameness. In the PBS group, seven out of nine animals experienced joint swelling, albeit smaller in size than the ASFV-Kenya-IX-1033-∆CD2v-inoculated group, of which two required treatment due to lameness.

Furthermore, the weight gain in the 28 days following inoculation with ASFV-Kenya-IX-1033-∆CD2v (2.2 kg ± 2.9) was reduced compared to the PBS group (4.3 kg ± 2.9), although this was not statistically significant due to the high variation in weight gain between pigs (data not shown).

### 3.2. Survival and Clinical Signs after Challenge with ASFV-Kenya-IX-1033

After challenge, PBS mock-immunised animals all developed clinical signs compatible with acute ASF and met the humane endpoint criteria between 4 and 8 days post challenge. In contrast, among the animals immunised with ASFV-Kenya-IX-1033-ΔCD2v, only one animal met the humane endpoint criteria at 9 days post challenge, with the remaining animals surviving until the end of the study (Figure 1b). Three of the surviving animals had a fever (rectal temperature ≥39.5 °C) and the other four animals appeared clinically normal after challenge; they did not have fever, had a normal appetite and were active throughout the 20-day follow-up period.

### 3.3. Immune Responses after Immunisation with ASFV-Kenya-IX-1033-∆CD2v

**Figure 2:**
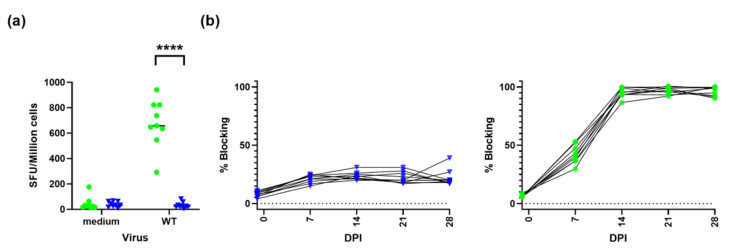
Immune responses after immunisation with ASFV-Kenya-IX-1033-∆CD2v. (**a**) IFN-γ ELISPOT using PBMC isolated from animals at day 21 after immunisation with ASFV-Kenya-IX-1033-ΔCD2 (green) or PBS (blue) and stimulation with either medium or ASFV-Kenya-IX-1033 wildtype at MOI of 0.1 (WT). Statistical analysis was carried out using multiple t-test. Asterisks indicate the statistical difference between experimental groups (**** *p* < 0.0001). (**b**) Percentage blocking in competitive p72 antibody ELISA in animals after mock immunisation with PBS (blue) or ASFV-Kenya-IX-1033-ΔCD2 (green).

Humoral and cellular immunity induced by ASFV-Kenya-IX-1033-∆CD2v were assessed by IFN-γ ELISpot and competitive p72 ELISA (Figure 2). There was a detectable IFN-γ response in all animals inoculated with ASFV-Kenya-IX-1033-∆CD2v using PBMC isolated at day 21 and in vitro stimulation with ASFV-Kenya-IX-1033. The magnitude of the IFN-γ responses varied between 292 and 941 SFU per million cells depending on the animal (Figure 2a). The humoral immune response was more uniform, with a 100% blocking in the competitive p72 ELISA for all animals immunised with ASFV-Kenya-IX-1033-∆CD2v from 14 days after challenge (Figure 2b).

### 3.4. Viral Titres after Immunisation with ASFV-Kenya-IX-1033-∆CD2v and Challenge with ASFV-Kenya-IX-1033

**Figure 3:**
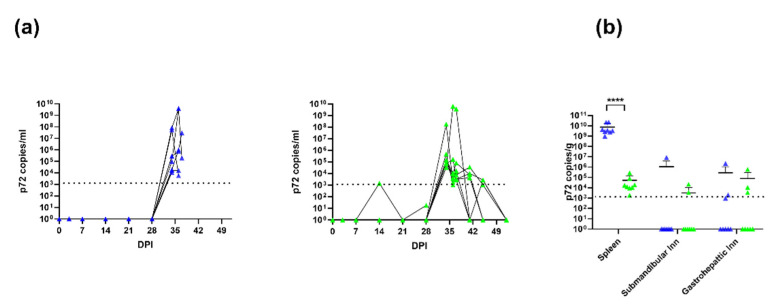
ASFV genome copies in blood and tissues. (**a**) p72 copies in EDTA blood after immunisation with PBS (blue) or ASFV-Kenya-IX-1033-ΔCD2 (green) and challenge with ASFV-Kenya-IX-1033. (**b**) p72 copies in spleen, submandibular lymph node and gastrohepatic lymph node of the eight animals challenged with ASFV-Kenya-IX-1033. The lower limit of detection of the qPCR was 1250 genome copies/mL or 1250 genome copies/g. When copy numbers were under 1250 genome copies/mL or g or when no Ct value was determined during qPCR, the value was registered as 10^0^ copies/mL. Statistical analysis was carried out using multiple t-test. Asterisks indicate the statistical difference between experimental groups (**** *p* < 0.0001).

Except for one time point in one animal, the p72 copy number, as assessed by qPCR, was below the lower limit of detection (1250 copies/mL) in the blood of animals immunised with ASFV-Kenya-IX-1033-∆CD2v. Post challenge, the p72 copy number increased in all animals with peaks in the ASFV-Kenya-IX-1033-∆CD2v-immunised animals between 1.5 × 10^5^ and 3.9 × 10^9^ copies per ml of blood and between 2.8 × 10^5^ and 3.9 × 10^9^ copies per ml of blood for the PBS immunised animals (Figure 3a). Thus, immunisation with ASFV-Kenya-IX-1033-∆CD2v does not appear to prevent the replication of the virus after challenge with ASFV-Kenya-IX-1033. However, the p72 copy numbers in the spleen were significantly lower in the ASFV-Kenya-IX-1033-∆CD2v-immunised group (Figure 3b). Most animals in the ASFV-Kenya-IX-1033-∆CD2v survived until the end of the study and were clinically normal at the time of euthanisation. In contrast, the PBS-immunised animals exhibited severe clinical signs and were euthanised after 4–8 days due to meeting the humane endpoint criteria.

### 3.5. Gross Pathological Lesions after Immunisation with ASFV-Kenya-IX-1033-∆CD2v and Challenge with ASFV-Kenya-IX-1033

**Figure 4:**
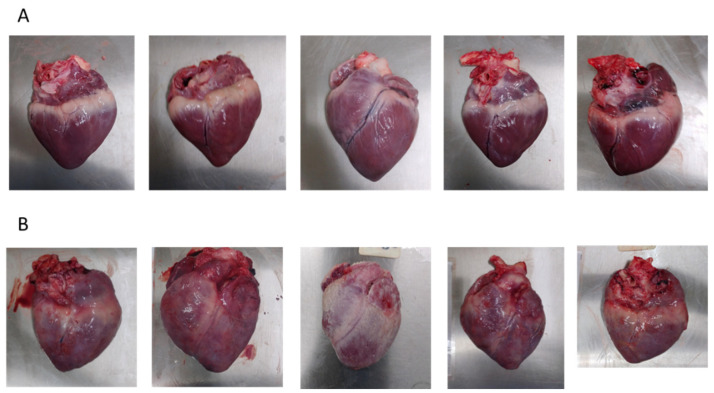
Gross-pathology after immunisation with ASFV-Kenya-IX-1033-ΔCD2 and challenge with ASFV-Kenya-IX-1033. (**A**) Representative pictures of hearts of pigs after mock immunisation with PBS and challenge with ASFV-Kenya-IX-1033. (**B**) Representative pictures of hearts of pigs after immunisation with ASFV-Kenya-IX-1033-ΔCD2 and challenge with ASFV-Kenya-IX-1033.

Animals were euthanised either when the human endpoint criteria were met or at the end of the study (Day 51). A post-mortem investigation was performed to assess whether gross pathology lesions were present. All animals in the mock-immunised group and challenged with ASFV-Kenya-IX-1033 exhibited gross pathological signs consistent with acute ASFV infection; lymph nodes were enlarged and either marbled, had haemorrhages, or had a blood clot-like appearance. Mild to moderate lung oedema was also observed. In contrast, animals inoculated with ASFV-Kenya-IX-1033-∆CD2v had lymph nodes without haemorrhages or a blood clot-like appearance. Six out of nine animals inoculated with ASFV-Kenya-IX-1033-∆CD2v had mild to severe fibrinous pericarditis at post-mortem investigation, whereas no pericarditis was observed in the PBS group (Figure 4). This suggests that the pericarditis is related to the infection with ASFV-Kenya-IX-1033-∆CD2v.

## 4. Discussion

The CD2v gene was deleted by a double homologous recombination from the highly pathogenic ASFV-Kenya-IX-1033 isolate propagated on wild boar lung (WSL) cells [24]. The aim of this study was to investigate the effect of this deletion on the virulence of the virus, and whether the CD2v-deleted virus induced protection against challenge with the parental virus. The virulence of ASFV-Kenya-IX-1033-ΔCD2 was significantly reduced in vaccinated pigs compared to the parental virus [21], with all animals surviving after immunisation. The CD2v gene was selected for deletion to generate an ASFV strain with reduced virulence, since some low-virulence ASFV isolates are non-haemadsorbing and have a truncated or deleted CD2v protein [17,18]. Furthermore, the experimental deletion of CD2v from the virulent BA71 (p72 genotype I) strain resulted in complete attenuation [19], whereas the deletion of CD2v (8-DR) in the Malawian strain Lil-20/1 and the Georgia strain (p72 genotype II) did not reduce virulence in vivo and resulted in a limited reduction in viraemia following challenge [12,20].

Petrovan et al. deleted CDV2v (locus EP402R) from the genome of Benin∆DP148R. This led to a reduction in virulence as compared to Benin∆DP148R. However, there was still residual virulence as pigs had transient increased rectal temperatures above 40.5 °C, lethargy and reduced appetite [11]. The effect of CD2v deletion from ASFV-Kenya-IX-1033 thus seems most similar to the deletion of CD2v from Benin∆DP148R, with a reduction in mortality and severity of clinical symptoms but retention of a degree of virulence. Thus, the development of LA-ASFV vaccines using different genotypes/strains appears to be more complicated than simply introducing the same gene deletion to a particular viral strain. A gene deletion can lead to complete attenuation, partial attenuation, or no attenuation depending on the specific target strain into which it is introduced. It will be important for the future development of LA-ASFV vaccines to understand the reason for these differences in attenuation.

Non-haemadsorbing naturally attenuated ASFV strains may have additional genomic changes; therefore, the reduced virulence might not be fully attributable to the changes in the CD2v locus alone but could be due to other genomic changes or a combination of the deletion/disruption of CD2v and other genomic changes. For CD2v-knockout strains generated by genome editing techniques, the genome changes other than the targeted deletion are expected to be limited, but for non-adsorbing naturally attenuated ASFV strains, changes may be present elsewhere in the genome, such as in the multigene families (MGF). In the case of the CD2v deletion mutants generated by genome editing techniques, whole-genome sequence information was not included in the publications describing Malawi Lil-20/1∆8-DR [12] and Benin∆DP148R∆EP402R [11] and the presence of undesired mutations can therefore not be excluded. However, targeted sequencing across the site of deletion was performed to confirm the deletion and the site of reporter gene insertion in the case of the mutated Benin virus [11]. No undesired genomic changes were detected in Georgia∆CD2v [20], whereas for the BA71 strain seven point mutations were detected, of which one was a non-synonymous change in the open-reading frame of the D250R gene leading to an amino acid substitution from histidine to tyrosine [19]. For ASFV-Kenya-IX-1033-ΔCD2, four SNPs were detected compared to ASF-Kenya-IX-1033 [21]. Two were previously described as SNPs between the macrophage-grown and WSL-grown ASF-Kenya-IX-1033 and thus are likely to have occurred prior to the introduction of the gene deletion. These comprise a non-synonymous SNP in g5R and a synonymous SNP in k11L. Of note, both the macrophage and WSL-grown stocks of ASFV-Kenya-IX-1033 are highly pathogenic, so these SNPs do not influence the virulence of ASFV-Kenya-IX-1033 [21]. In addition, two synonymous SNPs were detected in the j15R gene of the CD2v deletion mutant.

Despite the 100% survival rate after inoculation with ASFV-Kenya-IX-1033-∆CD2v, the strain had residual virulence when inoculated in pigs. The animals exhibited intermittent fever, joint swelling, and some had periodic lethargy. On post-mortem investigation, marbled lymph nodes and fibrinous pericarditis were seen in most animals, whereas pericarditis was not observed in the PBS-inoculated group, suggesting that ASFV-Kenya-IX-1033-∆CD2v is responsible for the observed pericarditis. Fibrinous pericarditis is described after chronic infection following infection with several low-virulence ASFV strains [17,26,27,28]. However, animals immunised with Benin∆DP148R∆EP402R, which had an additional deletion to CD2v, did not have any macroscopic lesions except for lympho-adenopathy [11]. No details with regard to macroscopic lesions after immunisation with BA71∆CD2v are provided in the manuscript [19]. In human medicine, idiopathic viral infections are believed to be responsible for 80–90% of pericarditis cases, including viruses such as Coxsackie B virus, HIV and influenza [29].

Although the cause of pericarditis is likely to be ASFV, it cannot be ruled out that the pigs had an underlying condition which presented due to immune suppression caused by ASFV-Kenya-IX-1033-∆CD2v inoculation. *Haemophilus parasuis* (Glasser disease), *Streptococcus suis*, *Mycoplasma hyopneumoniae* (Enzootic pneumonia) and *mycoplasma hyorhinus* are known to cause fibrinous pericarditis in domestic pigs. Limited information on the prevalence of these pathogens on the African continent is available. However, Dione et al. found seroprevalences for *Mycoplasma hyopneumonia* in Masaka and Lira districts in Uganda of 10.1% and 20.9%, respectively, and 68.2% and 73%, respectively, for *Streptococcus suis* [30,31]. Pigs are thus likely to be exposed to/infected with other pathogens, which could influence the success of LA-ASFV when deployed in the field. Even though the animals were apparently health, the animals included in this study were only tested for the presence/absence of ASFV by qPCR and seronegativity for ASFV by ELISA. The presence of co-infections in these pigs can therefore not be excluded.

The immunity induced after inoculation with ASFV-Kenya-IX-1033-∆CD2v protected 87.5% of the animals against mortality following challenge with virulent parental ASFV. All animals developed detectable immune responses to ASFV; 100% blocking was seen using the competitive p72 antibody ELISA from 2 weeks after inoculation, and strong ASFV-specific IFN-γ responses were detected using ELISpot. However, the one immunised animal that met the humane endpoint criteria had immune responses similar to the protected animals, suggesting that additional responses may also be important. Although immunity induced by ASFV-Kenya-IX-1033-∆CD2v prevented mortality after parental ASFV challenge, it did not prevent the development of clinical symptoms and macroscopic lesions consistent with chronic ASFV infection [26]. Reis et al. investigated antibody responses after infection with the low virulence ASFV/NH/P68 strain against different ASFV recombinant proteins and compared the responses of asymptomatic and chronically infected animals. For the proteins NP419L/DNA ligase, CP312R, B646L/p73, K196R/thymidine kinase and K205R, the antibody titres were significantly higher in animals that developed lesions [32]. It is, therefore, possible that some of the immune responses induced by ASFV-Kenya-IX-1033-∆CD2v may contribute to the development of chronic ASF. A more in-depth immunological investigation of both the cellular and humoral responses is required to shed further light on the precise role of different components of the immune response.

The deletion of CD2v from certain ASFV strains has resulted in LA-ASFV that cause low or no mortality and induce protection against pathogenic ASFV challenge. The attenuated BA71ΔCD2v virus protected against homologous and heterologous challenge in a dose-dependent manner. The highest doses used, 3.3 × 10^4^ PFU and 1 × 10^6^ PFU, protected against homologous challenge with BA71 and heterologous challenge with E75 and the Georgia strain [19]. Similarly, all animals surviving immunisation with Benin∆DP148R∆EP402R were protected upon challenge with the parental Benin strain [11]. For ASFV-Kenya-IX-1033-∆CD2v, partial protection (87.5%) against parental ASFV-Kenya-IX-1033 was observed. For Benin∆DP148R∆EP402R and ASFV-Kenya-IX-1033-∆CD2v, protection against strains other than the parental strain was not tested. In future, it will be important to know if strains can protect against more distant ASF viruses, especially in sub-Saharan Africa, where multiple ASFV strains circulate simultaneously in certain regions.

## Data Availability

Not applicable.

## References

[1] Qu H., Ge S., Zhang Y., Wu X., Wang Z. (2022). A Systematic Review of Genotypes and Serogroups of African Swine Fever Virus. Virus Genes.

[2] Animal Health Status of Regions. https://www.aphis.usda.gov/aphis/ourfocus/animalhealth/animal-and-animal-product-import-information/animal-health-status-of-regions.

[3] Vietnam Officially Announces Successful Production of a Vaccine against African Swine Fever. https://Vietnamagriculture.Nongnghiep.vn/Vietnam-Officially-Announces-Successful-Production-of-a-Vaccine-against-African-Swine-Fever-D324623.Html.

[4] Gladue D.P., Borca M.V. (2022). Recombinant ASF Live Attenuated Virus Strains as Experimental Vaccine Candidates. Viruses.

[5] Sánchez E.G., Riera E., Nogal M., Gallardo C., Fernández P., Bello-Morales R., López-Guerrero J.A., Chitko-Mckown C.G., Richt J.A., Revilla Y. (2017). Phenotyping and Susceptibility of Established Porcine Cells Lines to African Swine Fever Virus Infection and Viral Production. Sci. Rep..

[6] Krug P.W., Holinka L.G., O’Donnell V., Reese B., Sanford B., Fernandez-Sainz I., Gladue D.P., Arzt J., Rodriguez L., Risatti G.R. (2015). The Progressive Adaptation of a Georgian Isolate of African Swine Fever Virus to Vero Cells Leads to a Gradual Attenuation of Virulence in Swine Corresponding to Major Modifications of the Viral Genome. J. Virol..

[7] Borca M.V., Holinka L.G., Berggren K.A., Gladue D.P. (2018). CRISPR-Cas9, a Tool to Efficiently Increase the Development of Recombinant African Swine Fever Viruses. Sci. Rep..

[8] Abkallo H.M., Svitek N., Oduor B., Awino E., Henson S.P., Oyola S.O., Mwalimu S., Assad-Garcia N., Fuchs W., Vashee S. (2021). Rapid CRISPR/Cas9 Editing of Genotype IX African Swine Fever Virus Circulating in Eastern and Central Africa. Front. Genet..

[9] Reis A.L., Abrams C.C., Goatley L.C., Netherton C., Chapman D.G., Sanchez-Cordon P., Dixon L.K. (2016). Deletion of African Swine Fever Virus Interferon Inhibitors from the Genome of a Virulent Isolate Reduces Virulence in Domestic Pigs and Induces a Protective Response. Vaccine.

[10] Revilla Y., Pérez-Núñez D., Richt J.A. (2018). African Swine Fever Virus Biology and Vaccine Approaches. Adv. Virus Res..

[11] Petrovan V., Rathakrishnan A., Islam M., Goatley L.C., Moffat K., Sanchez-Cordon P.J., Reis A.L., Dixon L.K. (2022). Role of African Swine Fever Virus Proteins EP153R and EP402R in Reducing Viral Persistence in Blood and Virulence in Pigs Infected with BeninΔDP148R. J. Virol..

[12] Borca M.V., Carrillo C., Zsak L., Laegreid W.W., Kutish G.F., Neilan J.G., Burrage T.G., Rock D.L. (1998). Deletion of a CD2-like Gene, 8-DR, from African Swine Fever Virus Affects Viral Infection in Domestic Swine. J. Virol..

[13] Rodríguez J.M., Yáñez R.J., Almazán F., Viñuela E., Rodriguez J.F. (1993). African Swine Fever Virus Encodes a CD2 Homolog Responsible for the Adhesion of Erythrocytes to Infected Cells. J. Virol..

[14] Goatley L.C., Dixon L.K. (2011). Processing and Localization of the African Swine Fever Virus CD2v Transmembrane Protein. J. Virol..

[15] Alejo A., Matamoros T., Guerra M., Andrés G. (2018). A Proteomic Atlas of the African Swine Fever Virus Particle. J. Virol..

[16] Dixon L.K., Islam M., Nash R., Reis A.L. (2019). African Swine Fever Virus Evasion of Host Defences. Virus Res..

[17] Boinas F.S., Hutchings G.H., Dixon L.K., Wilkinson P.J. (2004). Characterization of Pathogenic and Non-Pathogenic African Swine Fever Virus Isolates from Ornithodoros Erraticus Inhabiting Pig Premises in Portugal. J. Gen. Virol..

[18] Gallardo C., Sánchez E.G., Pérez-Núñez D., Nogal M., de León P., Carrascosa Á.L., Nieto R., Soler A., Arias M.L., Revilla Y. (2018). African Swine Fever Virus (ASFV) Protection Mediated by NH/P68 and NH/P68 Recombinant Live-Attenuated Viruses. Vaccine.

[19] Monteagudo P.L., Lacasta A., López E., Bosch L., Collado J., Pina-pedrero S., Correa-fiz F., Accensi F., Navas J.M., Vidal E. (2017). BA71∆CD2v: A new recombinant live attenuated African swine fever virus with cross-Protective Capabilities. J. Virol..

[20] Borca M.V., Donnell V.O., Holinka L.G., Risatti G.R., Ramirez-Medina E., Vuono E., Shi J., Pruitt S., Ray A., Silva E. (2020). Deletion of CD2-like Gene from the Genome of African Swine Fever Virus Strain Georgia Does Not Attenuate Virulence in Swine. Scientific.

[21] Hemmink J.D., Abkallo H.M., Henson S.P., Khazalwa E.M., Oduor B., Lacasta A., Okoth E., Riitho V., Fuchs W., Bishop R.P. (2022). The African Swine Fever Isolate ASFV-Kenya-IX-1033 is Highly Virulent and Stable after Propagation in the Wild Boar Cell Line WSL. Viruses.

[22] Onzere C.K., Bastos A.D., Okoth E.A., Lichoti J.K., Bochere E.N., Owido M.G., Ndambuki G., Bronsvoort M., Bishop R.P. (2018). Multi-Locus Sequence Typing of African Swine Fever Viruses from Endemic Regions of Kenya and Eastern Uganda (2011–2013) Reveals Rapid B602L Central Variable Region Evolution. Virus Genes.

[23] King K., Chapman D., Argilaguet J.M., Fishbourne E., Hutet E., Cariolet R., Hutchings G., Oura C.A.L., Netherton C.L., Moffat K. (2011). Protection of European Domestic Pigs from Virulent African Isolates of African Swine Fever Virus by Experimental Immunisation. Vaccine.

[24] Hübner A., Petersen B., Keil G.M., Niemann H., Mettenleiter T.C., Fuchs W. (2018). Efficient Inhibition of African Swine Fever Virus Replication by CRISPR/Cas9 Targeting of the Viral P30 Gene (CP204L). Sci. Rep..

[25] King D.P., Reid S.M., Hutchings G.H., Grierson S.S., Wilkinson P.J., Dixon L.K., Bastos A.D.S., Drew T.W. (2003). Development of a TaqMan^®^ PCR Assay with Internal Amplification Control for the Detection of African Swine Fever Virus. J. Virol. Methods.

[26] Sanchez-Cordon P., Vidaña B., Neimanis A., Núñez A., Wikström E., Iacolina L., Penrith M.-L., Bellini S., Chenais E., Jori F., Montoya M., Ståhl K., Gavier-Widén D. (2021). Understanding and Combatting African Swine Fever.

[27] Sánchez-Cordón P.J., Chapman D., Jabbar T., Reis A.L., Goatley L., Netherton C.L., Taylor G., Montoya M., Dixon L. (2017). Different Routes and Doses Influence Protection in Pigs Immunised with the Naturally Attenuated African Swine Fever Virus Isolate OURT88/3. Antivir. Res..

[28] Sánchez-Cordón P.J., Jabbar T., Chapman D., Dixon L.K., Montoya M. (2020). Absence of Long-Term Protection in Domestic Pigs Immunized with Attenuated African Swine Fever Virus Isolate OURT88/3 or BeninΔMGF Correlates with Increased Levels of Regulatory T Cells and Interleukin-10. J. Virol..

[29] Imazio M., Gaita F., LeWinter M. (2015). Evaluation and Treatment of Pericarditis: A Systematic Review. JAMA J. Am. Med. Assoc..

[30] Dione M., Masembe C., Akol J., Amia W., Kungu J., Lee H.S., Wieland B. (2018). The Importance of On-Farm Biosecurity: Sero-Prevalence and Risk Factors of Bacterial and Viral Pathogens in Smallholder Pig Systems in Uganda. Acta Trop..

[31] Oba P., Wieland B., Mwiine F.N., Erume J., Gertzell E., Jacobson M., Dione M.M. (2020). Status and Gaps of Research on Respiratory Disease Pathogens of Swine in Africa. Porc. Health Manag..

[32] Reis A.L., Parkhouse R.M.E., Penedos A.R., Martins C., Leitáo A. (2007). Systematic Analysis of Longitudinal Serological Responses of Pigs Infected Experimentally with African Swine Fever Virus. J. Gen. Virol..

